# Estimated Zika virus importations to Europe by travellers from Brazil

**DOI:** 10.3402/gha.v9.31669

**Published:** 2016-05-17

**Authors:** Eduardo Massad, Ser-Han Tan, Kamran Khan, Annelies Wilder-Smith

**Affiliations:** 1 Department of Medicine, University of Sao Paolo, Sao Paolo, Brazil; 2 London School of Hygiene and Tropical Medicine, London, UK; 3 School of Computer Engineering, Nanyang Technological University, Singapore; 4 Li Ka Shing Knowledge Institute, St Michael's Hospital, Toronto, Canada; 5 Institute of Public Health, University of Heidelberg, Germany; 6 Department Public Health and Clinical Medicine, Epidemiology and Global Health, Umeå University, SE-901 85 Umeå, Sweden; 7 Lee Kong Chian School of Medicine, Nanyang Technological University, Singapore

**Keywords:** Zika virus, travel, importations, Brazil, Europe

## Abstract

**Background:**

Given the interconnectivity of Brazil with the rest of the world, Zika virus (ZIKV) infections have the potential to spread rapidly around the world via viremic travellers. The extent of spread depends on the travel volume and the endemicity in the exporting country. In the absence of reliable surveillance data, we did mathematical modelling to estimate the number of importations of ZIKV from Brazil into Europe.

**Design:**

We applied a previously developed mathematical model on importations of dengue to estimate the number of ZIKV importations into Europe, based on the travel volume, the probability of being infected at the time of travel, the population size of Brazil, and the estimated incidence of ZIKV infections.

**Results:**

Our model estimated between 508 and 1,778 imported infections into Europe in 2016, of which we would expect between 116 and 355 symptomatic Zika infections; with the highest number of importations being into France, Portugal and Italy.

**Conclusions:**

Our model identified high-risk countries in Europe. Such data can assist policymakers and public health professionals in estimating the extent of importations in order to prepare for the scale up of laboratory diagnostic assays and estimate the occurrence of Guillain–Barré Syndrome, potential sexual transmission, and infants with congenital ZIKV syndrome.

## Introduction

In May 2015, an outbreak of Zika virus (ZIKV) infections was first reported in Brazil, and by December 2015, 500,000–1,500,000 ZIKV infections were estimated (1). By October 2015, increasing number of microcephaly cases and other neonatal malformations were thought to be associated with ZIKV infections (2). On 1 February 2016, the clusters of microcephaly and Guillain–Barré Syndrome (GBS) cases in likely association with ZIKV infections were declared a public health emergency of international concern (3). Given the interconnectivity of Brazil with the remainder of the world, ZIKV has the potential to spread rapidly around the world via viremic travellers (4). The extent of spread depends on the travel volume to destination countries and the endemicity in the exporting country (5–7). Because of the mild clinical manifestations of the disease in the vast majority of cases, ZIKV infections in individual travellers are unlikely to lead to cancellation of flights or disruption of holiday/business plans. Furthermore, 80% of all infections are thought to be asymptomatic. The biggest concern is the spread to areas where suitable mosquito vectors exist and importation could trigger further outbreaks. However, given that sexual transmission of ZIKV has been reported, the spread of ZIKV via viremic travellers to areas without the *Aedes* mosquitoes is equally of concern (8). Sexual transmission to non-travelling contacts in Europe could propagate ZIKV infections in Europe, resulting in a potential upsurge of GBS cases as a result of imported ZIKV infections and putting pregnant women at risk. Therefore it is important to estimate the potential number of travellers returning to Europe with ZIKV infections.

ZIKV infections remain underdiagnosed and underreported because of the non-specific and mild manifestations and lack of widely available diagnostic assays. Therefore, for the time being any estimates on the epidemiological burden remain crude estimates. We based our calculations on the published estimate of 500,000–1,500,000 infections (both symptomatic and asymptomatic) for the year 2015 in Brazil (1). Reliance on reported events of importation will only underestimate the true importation risk as most imported cases will not be detected and reported, unless the clinical manifestations are more severe. In the absence of reliable surveillance data, mathematical modelling is necessary to estimate the number of importations of ZIKV from Brazil into Europe.

## Methods

We applied a previously developed mathematical model on exportations to estimate the number of ZIKV importations into Europe (9). This model takes into account the travel volume, the probability of being infected at the time of travel, the population size of Brazil, and the estimated incidence of ZIKV infections (estimated numbers over population size). The model was previously developed to estimate the risk of dengue acquisition in international travellers (10–12), and has also been applied to estimate polio virus importations (13).

The number of travellers departing from Brazilian airports on commercial flights to each of the European countries was obtained from the International Air Transport Association (IATA) for the year 2012. As we only had access to the year 2012 flight data, the travel pattern of outgoing flights in 2015 or 2016 was assumed to not have changed significantly.

We calculated the force of infection, *λ*(*t*) from the assumption that there had been 0.5 to 1.5 million ZIKV infections in Brazil. In addition, we assumed that the seasonal distribution of cases followed the same as for dengue, given that both viral infections share the same *Aedes* vectors, and initial observations have claimed that ZIKV seems to follow the path of dengue (14). As populations of *Aedes aegypti* and *Aedes albopictus* are climate sensitive and display a seasonal pattern in Brazil (15–17), ZIKV infections are likely to exhibit the same seasonal pattern as dengue in Brazil.

The steps for the mathematical models are detailed in the Supplement. In brief, we first fitted a continuous function to the time distribution of notified cases from which we estimate the force of infection *λ*(*t*). The product of the force of infection by the fraction of susceptible individuals is the number of reported cases.

The individual risk of acquiring the infection from the ZIKV-infected mosquitoes, *Risk* (*t*), is given by

1 $$Risk(t)=1-\exp(-\int_{t_{1}}^{t_{2}}\lambda (t)dt)$$

where, again, *λ*(*t*) is the force of infection or incidence density rate; *t*_1_ is, in the case of travellers, the moment they arrive at the endemic area; and *t*_2_ is the moment they depart. Note that the concept of risk expressed in equation (1) means the risk for travellers that remain in the ZIKV endemic area for the period between *t*_1_ and *t*_2_. For locals, *t*_2_–*t*_1_ is the time interval considered for the risk calculation (e.g. the month-by-month risk calculation).

**Fig. 1:**
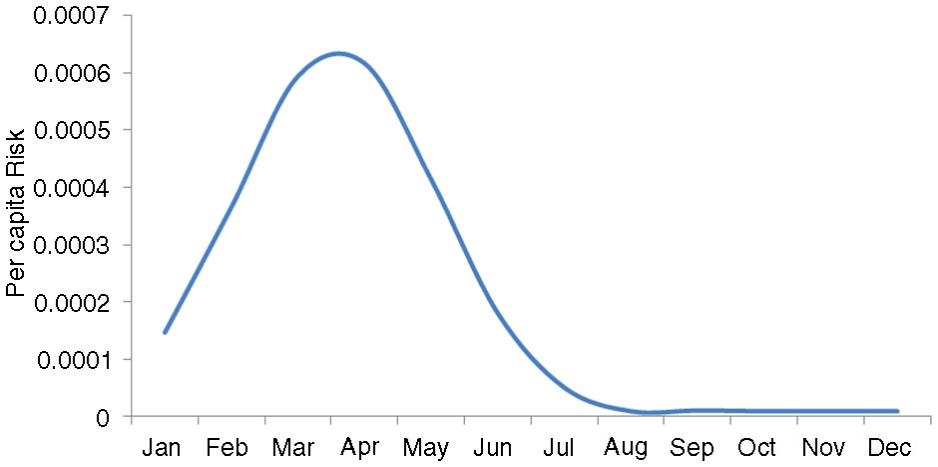
Calculated per capita risk (probability of being infected). *Risk*(*t*)=1–exp[−*λ*(*t*)*t*], where *λ*(*t*) is the force of infection.

The risk varies with time. As Fig. 1 shows, this risk is highest in the months with the highest number of reported dengue cases (as a consequence of a higher density of infected mosquitoes), at its maximum by the month of April. This would also fit with the observation of the onset of excess microcephaly cases in October 2015 (6–9 months after the high season of January to April).

As the function *Risk* (*t*) represents the individual risk of acquiring the infection, we can use it as the probability that one passenger flying from a Brazilian airport is infected with the ZIKV. By multiplying the individual probability of being infected by the number of passengers leaving Brazilian airports, we have the total number of expected infections that are flying to European countries.

Our model applies to individuals from Brazil travelling to Europe or travellers having visited Brazil and now returning to Europe.

## Results

**Fig. 2:**
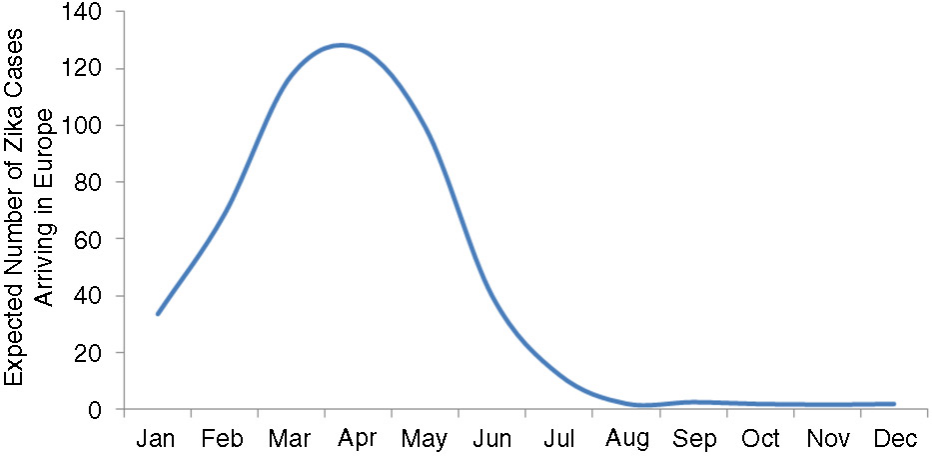
Expected number of travellers with Zika virus infections arriving in Europe by month, in the year 2015.

**Fig. 3:**
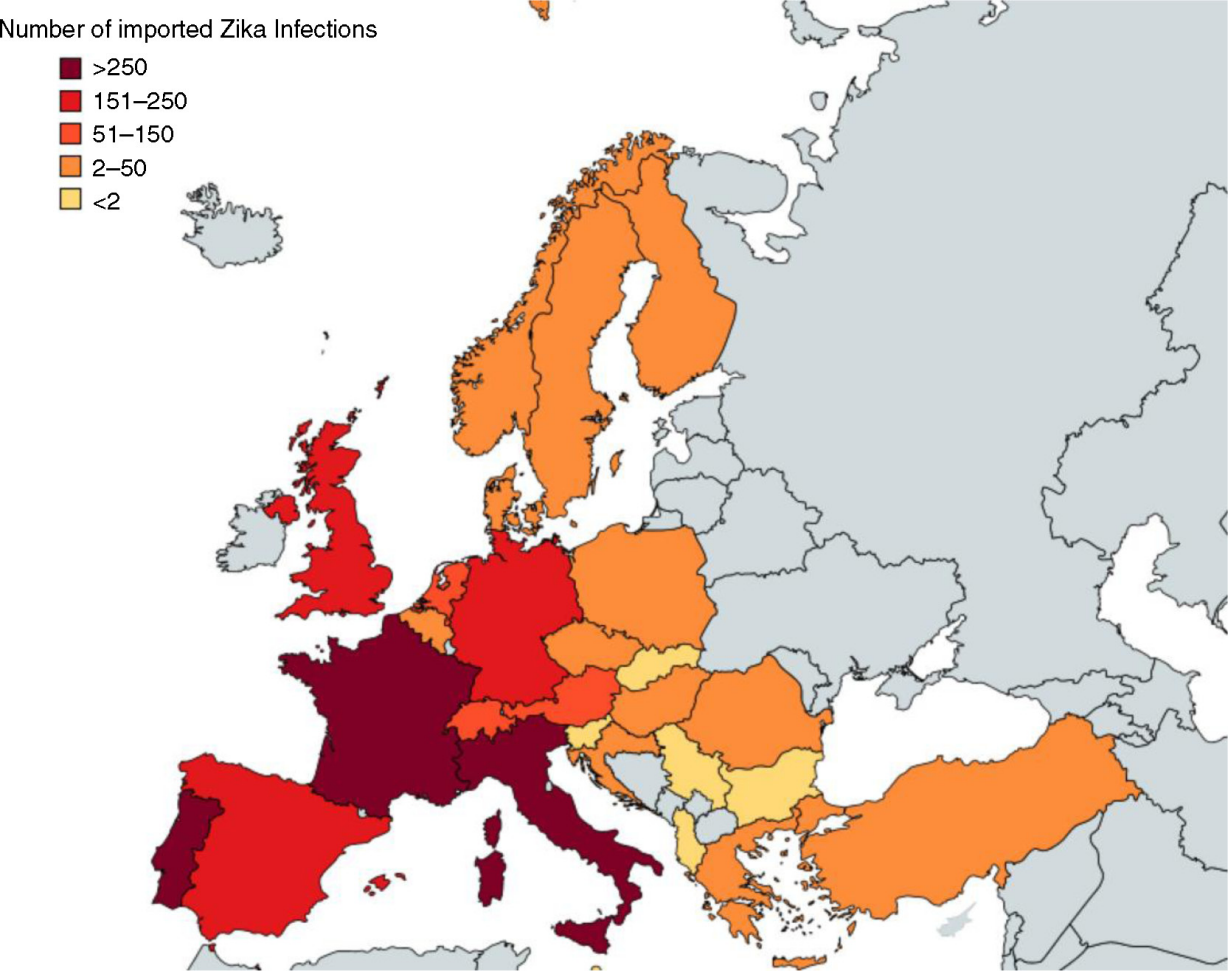
Estimated importations of Zika virus infections via travellers from Brazil to Europe in the year 2015, based on a high estimate of 1,500,000 Zika virus infections in Brazil.

Figures 1 and 2 show the resulting curve for the individual risk of acquiring the infection and the expected number of ZIKA cases arriving in Europe by month, respectively. Table 1 and the Map show the results of the expected number of ZIKV cases exported to European countries from Brazil, based on an estimated lower bound of 500,000 and upper bound of 1.5 million ZIKV infections, respectively, assuming that these ZIKV infections exhibit the same seasonal pattern as dengue infections. In total, our models estimated between 508 and 1,778 imported cases, respectively, into all European countries, with the highest numbers being in France, Portugal, and Italy (Table 1 and Fig. 3). Of these, 80% would likely be asymptomatic; hence, we would expect between 116 and 355 symptomatic ZIKV infections.

**Table 1:** Estimated imported ZIKV infections from Brazil to Europe based on the 1.5 million and 500,000 ZIKV infections scenarios in 2015

		Jan	Feb	Mar	Apr	May	Jun	Jul	Aug	Sep	Oct	Nov	Dec	Total
Individual risk for each scenario	500 K	0.000146	0.000366	0.000594	0.000617	0.000414	0.00018	5.03E-05	9.06E-06	1.05E-05	9.06E-06	9.06E-06	9.06E-06	
	1.5 G	0.000511	0.00128	0.00208	0.00216	0.00145	0.000631	0.000176	3.17E-05	3.67E-05	3.17E-05	3.17E-05	3.17E-05	
Albania	Travellers	9	12	8	9	10	67	1	28	9	13	21	0	187
Expected cases for each scenario	500 K	0	0	0	0	0	0	0	0	0	0	0	0	0
	1.5 G	0	0	0	0	0	0	0	0	0	0	0	0	0
Austria	Travellers	1986	1840	2570	2203	24000	2304	2339	3235	3660	2067	2245	0	48449
Expected cases for each scenario	500 K	0	1	2	1	10	0	0	0	0	0	0	0	14
	1.5 G	1	2	5	5	35	1	0	0	0	0	0	0	51
Belgium	Travellers	2733	2543	2565	2647	2375	2599	3194	3513	2313	2296	1881	2449	31108
Expected cases for each scenario	500 K	0	1	2	2	1	0	0	0	0	0	0	0	6
	1.5 G	1	3	5	6	3	2	1	0	0	0	0	0	22
Bulgaria	Travellers	156	124	67	122	223	159	159	319	69	92	76	158	1724
Expected cases for each scenario	500 K	0	0	0	0	0	0	0	0	0	0	0	0	0
	1.5 G	0	0	0	0	0	0	0	0	0	0	0	0	1
Croatia	Travellers	229	271	234	354	491	926	675	597	883	551	201	200	5612
Expected cases for each scenario	500 K	0	0	0	0	0	0	0	0	0	0	0	0	1
	1.5 G	0	0	0	1	1	1	0	0	0	0	0	0	3
Czech Rep	Travellers	561	901	1096	1092	1945	1663	1867	1805	2438	1470	1193	1026	17057
Expected cases for each scenario	500 K	0	0	1	1	1	0	0	0	0	0	0	0	3
	1.5 G	0	1	2	2	3	1	0	0	0	0	0	0	11
Denmark	Travellers	3384	2151	1878	2015	2880	3274	4052	4421	1971	1506	1531	1725	30788
Expected cases for each scenario	500 K	0	1	1	1	1	1	0	0	0	0	0	0	6
	1.5 G	2	3	4	4	4	2	1	0	0	0	0	0	20
Finland	Travellers	1405	799	651	687	833	1026	843	660	374	537	661	0	8476
Expected cases for each scenario	500 K	0	0	0	0	0	0	0	0	0	0	0	0	2
	1.5 G	1	1	1	1	1	1	0	0	0	0	0	0	7
France	Travellers	29302	28118	33352	32796	35533	37932	40265	32572	37959	33476	27270	39248	407823
Expected cases for each scenario	500 K	4	10	20	20	15	7	2	0	0	0	0	0	80
	1.5 G	15	36	69	71	52	24	7	1	1	1	1	1	279
Germany	Travellers	21552	20305	22563	21564	21872	20226	21372	24965	29920	22872	21713	23703	
Expected cases for each scenario	500 K	3	7	13	13	9	4	1	0	0	0	0	0	
	1.5 G	11	26	47	47	32	13	4	1	1	1	1	1	
Greece	Travellers	712	590	1499	1141	1484	1805	2329	1836	2439	1386	646	690	16557
Expected cases for each scenario	500 K	0	0	1	1	1	0	0	0	0	0	0	0	3
	1.5 G	0	1	3	2	2	1	0	0	0	0	0	0	11
Hungary	Travellers	472	553	531	714	1190	1244	1437	1617	1282	788	452	465	10745
Expected cases for each scenario	500 K	0	0	0	0	0	0	0	0	0	0	0	0	2
	1.5 G	0	1	1	2	2	1	0	0	0	0	0	0	7
Italy	Travellers	36580	30257	34235	33521	38120	32883	35912	33365	39478	34887	28373	30136	407747
Expected cases for each scenario	500 K	5	11	20	21	16	6	2	0	0	0	0	0	83
	1.5 G	19	39	71	72	55	21	6	1	1	1	1	1	289
Malta	Travellers	97	67	61	65	127	86	102	96	73	51	114	108	1047
Expected cases for each scenario	500 K	0	0	0	0	0	0	0	0	0	0	0	0	0
	1.5 G	0	0	0	0	0	0	0	0	0	0	0	0	1
Netherland	Travellers	8055	6418	3727	7209	6997	7051	7669	7328	7006	7103	6391	6396	81350
Expected cases for each scenario	500 K	1	2	2	4	3	1	0	0	0	0	0	0	15
	1.5 G	4	8	8	16	10	4	1	0	0	0	0	0	53
Norway	Travellers	2423	1488	1409	2034	1728	1826	2479	1943	1677	1305	1354	2116	21782
Expected cases for each scenario	500 K	0	1	1	1	1	0	0	0	0	0	0	0	4
	1.5 G	1	2	3	4	3	1	0	0	0	0	0	0	15
Poland	Travellers	1016	760	884	1635	1344	1784	1828	1272	1018	1160	1025	971	14697
Expected cases for each scenario	500 K	0	0	1	1	1	0	0	0	0	0	0	0	3
	1.5 G	1	1	2	4	2	1	0	0	0	0	0	0	10
Portugal	Travellers	35079	29220	30250	34716	34795	35512	38984	34306	39754	30603	28926	33905	406050
Expected cases for each scenario	500 K	5	11	18	21	14	6	2	0	0	0	0	0	80
	1.5 G	18	37	63	75	50	22	7	1	1	1	1	1	278
Romania	Travellers	610	654	482	513	540	468	448	764	698	646	479	545	6847
Expected cases for each scenario	500 K	0	0	0	0	0	0	0	0	0	0	0	0	1
	1.5 G	0	1	1	1	1	0	0	0	0	0	0	0	5
Serbia	Travellers	124	69	83	64	119	115	74	140	57	140	74	75	1134
Expected cases for each scenario	500 K	0	0	0	0	0	0	0	0	0	0	0	0	0
	1.5 G	0	0	0	0	0	0	0	0	0	0	0	0	1
Slovakia	Travellers	11	5	8	5	13	14	28	3	20	9	11	3	130
Expected cases for each scenario	500 K	0	0	0	0	0	0	0	0	0	0	0	0	0
	1.5 G	0	0	0	0	0	0	0	0	0	0	0	0	0
Slovenia	Travellers	261	26	30	117	172	108	103	117	157	61	96	50	1298
Expected cases for each scenario	500 K	0	0	0	0	0	0	0	0	0	0	0	0	0
	1.5 G	0	0	0	0	0	0	0	0	0	0	0	0	1
Spain	Travellers	23205	22487	20259	21545	24095	25562	28821	20111	24405	21842	16803	22603	271738
Expected Cases for Each scenario	500 K	3	8	12	13	10	5	1	0	0	0	0	0	54
	1.5 G	12	29	42	47	35	16	5	1	1	1	1	1	189
Switzerland	Travellers	25562	11487	10804	8521	8295	9701	9461	11820	7604	9249	8803	11189	132496
Expected cases for each scenario	500 K	4	4	6	5	3	2	0	0	0	0	0	0	26
	1.5 G	13	15	22	18	12	6	2	0	0	0	0	0	90
Sweden	Travellers	3617	2458	2823	2082	1962	2310	2379	2015	1586	1428	1671	1576	25907
Expected cases for each scenario	500 K	1	1	2	1	1	0	0	0	0	0	0	0	6
	1.5 G	2	3	6	4	3	1	0	0	0	0	0	0	20
UK	Travellers	28678	22484	23246	24708	24263	25302	30344	24356	26938	22458	22068	23657	298502
Expected cases for each scenario	500 K	4	8	14	15	10	5	2	0	0	0	0	0	59
	1.5 G	15	29	48	53	35	16	5	1	1	1	1	1	206
Turkey	Travellers	1717	2258	2760	3890	4097	4432	4321	3641	5417	4394	2816	3691	43434
Expected cases for each scenario	500 K	0	1	2	2	2	1	0	0	0	0	0	0	8
	1.5 G	1	3	6	8	6	3	1	0	0	0	0	0	28
Total number of cases for each scenario	500 K													508
	1.5 G													1778

## Conclusions

Our estimates are consistent with those reported by the European Centre for Disease Control (ECDC). As of 3 March 2016, ECDC had recorded 209 imported cases into 16 European countries, of which 81 were into France, and 32 into Spain (18). Geographical distribution of ZIKV has steadily broadened since the virus was first detected in Brazil in 2015. By March 2016, ZIKV transmission had been reported in 28 countries and territories (19); hence, the exportation risk will be even higher than we reported. However, we were not able to calculate such a risk for the other countries as incidence data for those countries have not yet been published. Given that Brazil so far has been the country most affected with the highest absolute numbers of estimated ZIKV infections, it is justified to focus our model on Brazil as exporting country only, until more data are available from other Latin American countries.

A limitation of our study is that the underlying assumption of our model is the equal distribution of cases throughout the country, and the equal probability of travelling throughout the Brazilian population. However, in early 2015, the geographic concentration of most cases were in Northeast Brazil – but by late 2015 and early 2016, the distribution was already much wider spread with all major cities in Brazil being affected (18, 20–22). Hence our modelled estimates of ZIKV exportations based on travel volume will be a more accurate reflection of the situation in 2016, assuming that the year 2016 will also see between 500,000 and 1,500,000 ZIKV infections.

According to the French Polynesian case control study on ZIKV-related GBS, one would expect 24 GBS cases out of 100,000 ZIKV infections (23). In other words, if these estimates hold true, one would need to have 5,000 imported ZIKV infections to see one case of ZIKV-associated GBS in returning travellers from ZIKV-affected countries. Given the current exportation numbers estimated to be no more than 1,800, the probability of a ZIKV-associated GBS case in Europe in 2015 or 2016 is relatively low. However, the number of ZIKV-affected countries within Latin America, the Caribbean, and beyond is rising, and hence the likelihood of substantial number of returning travellers presenting with GBS is will increase. The true risk of ZIKV-related infections that can lead to central nervous system malformations and microcephaly in pregnant women is currently unknown, especially for sexual transmission (24). However, potentially every single viremic male returning traveller could infect his pregnant or non-pregnant partner, especially in the first 2–4 weeks after ZIKV infection (25–27). Hence, the Centre for Disease Control and travel medicine providers have advised for precautions (abstinence or condoms) to be taken for men returning home from ZIKV-affected countries, particularly in the first few weeks after return (28, 29).

An additional cause of concern is the risk of ZIKV establishing itself in European regions where the presence of *A. albopictus* is endemic, in particular for Mediterranean countries recording increasingly hotter summers (30), although the ZIKA competence for *A. albopictus* is not fully known at this stage.

We identified high-risk countries in Europe, and policymakers and clinicians need to be aware of such data. Furthermore, our models can be applied by individual countries or by continents alike and used as an additional tool to estimate the risk of importation based on the main contributing factors such as travel volume and the evolving ZIKA endemicity in exporting countries. Our models help policymakers estimate the extent of importations in order to prepare for the scale up of laboratory diagnostic assays and estimate the occurrence of GBS, potential sexual transmission, and infants with congenital ZIKA syndrome.

## Paper context

The Zika virus outbreak in Brazil has gripped the world's attention. We applied mathematical modelling to estimate the extent of Zika virus importations from Brazil to Europe for the year 2016. Our model estimated between 508 and 1,778 imported infections into Europe in 2016, with the highest number of importations being into France, Portugal, and Italy. Such data can assist policymakers to scale up preparatory measures in Europe.
